# Hydrogeochemical characteristics and air quality risks associated with gold mining operations in Egypt using geochemical modeling and risk indices

**DOI:** 10.1016/j.heliyon.2024.e31086

**Published:** 2024-05-22

**Authors:** Ahmed Ali El-Sayed M. Ata, Mobarak H. Aly, Hend Hussein, Mohamed Hamdy Eid, Mostafa R. Abukhadra, Ahmed M. El-Sherbeeny, Stefano Bellucci, Mohamed Gad

**Affiliations:** a Evaluation of Natural Resources Department, Environmental Studies and Research Institute (ESRI), University of Sadat City, Minufiya, 32897, Egypt; b Geology Department, Faculty of Science, Damanhour University, Damanhour, 22511, Egypt; c Institute of Environmental Management, Faculty of Earth Science, University of Miskolc, 3515, Miskolc, Hungary; d Geology Department, Faculty of Science, Beni-Suef University, Beni-Suef, 65211, Egypt; e Materials Technologies and their Applications Lab, Geology Department, Faculty of Science, Beni-Suef University, Beni-Suef City, Egypt; f Industrial Engineering Department, College of Engineering, King Saud University, P.O. Box 800, Riyadh, 11421, Saudi Arabia; g INFN, Laboratori Nazionali di Frascati, E. Fermi 54, 00044, Frascati, Italy

**Keywords:** Gold mining, Hazard index, Risk assessment, Water quality, Air quality

## Abstract

The success of industrial operations depends on the effective identification, appraisal, and mitigation of possible hazards and associated environmental concerns. This report provides a complete review of environmental management techniques at the Sukari Gold Mine (SGM), located in the southeastern desert of Egypt. Extensive environmental measurements were taken to assess air and water quality, identify hazards, and analyze risks on the SGM premises. Air quality and noise intensity levels were measured at 39 places around the mine's working region. The findings found noncompliance with the Egyptian Environmental Law's (EEL4/94) noise exposure limitations, with the Power Generator House having the maximum noise levels at 107 dB. Remedial measures such as personal protective equipment (PPE) and exposure limit reduction strategies are being considered to address elevated noise levels. Measurements of particulate matter (PM10) and noxious gases (e.g., CO, SO2, NO2, HCN, and NH3) were conducted in workplace and ambient environments. Elevated PM10 concentrations were particularly concerning in underground regions, forcing the deployment of water depression techniques and improved PPE measures. While gas emissions from most activities remained under regulatory limits, select zones showed hydrogen cyanide (HCN) levels that exceeded permitted thresholds, necessitating specific control actions. Using hazard index (HI) and risk rating assessments, this study found different risk profiles across SGM's workplaces, focusing on high-risk regions for focused intervention. Additionally, a water assessment near a Tailing Storage Facility (TSF) was conducted to monitor the impact of mining activities on groundwater quality. The study revealed that groundwater in the region belongs to the Na–K–Cl–SO_4_ and Ca–Mg–Cl–SO_4_ water classes, with potential degradation attributed to high mineralization processes induced by aquifer materials and seawater intrusion. The findings underscore the importance of ongoing monitoring, control measures, and implementation of programs to ensure environmental sustainability and minimize risks associated with mining activities in the Sukari Gold Mines. This research highlights the imperative of continuous monitoring, proactive control measures, and the implementation of environmental initiatives to ensure the sustainability of mining operations within the Sukari Gold Mines.

## 1 Introduction

Egypt boasts numerous mineral sites, including approximately 90 gold mines in the Eastern Desert with historical roots dating back to ancient times. The country is experiencing a significant effort in mining activities, particularly in gold mining. The SGM was selected to assess air and water quality, identify hazards, and analyze risks on the SGM premises where SGM is considered one of the largest in the Eastern Desert [1]. In many global economies, mineral and mining industries contribute to economic growth and production. The impact of gold mining on groundwater quality in the context of the Egyptian gold mining scheme has been scrutinized, with a particular focus on environmental repercussions, especially water supply depletion [2]. The Sukari gold mine is a significant asset in Egypt's mining landscape, with substantial ore reserves discovered through recent underground mapping, core drilling, and geochemical studies. Its ongoing development underscores its potential to contribute significantly to the country's gold extraction efforts in the coming years. The environmental effects of gold mining, particularly on groundwater quality, are a subject of extensive discussion. Factors such as recharge, aquifer compounds, residence time, leaching, mineral dissolution, and ion exchange contribute to the geochemical characteristics of groundwater [3,4]. Systematic groundwater assessment employing imitative techniques, including Piper trilinear diagram, Chadha diagram, and Gibbs diagram [[5], [6], [7]], provides insights into water quality control [[8], [9], [10], [11], [12], [13], [14]].

Water quality is assessed through physicochemical parameters representing groundwater characteristics, and various geochemical models (saturation index, evaporation, and mixing models) are employed for this purpose [[8], [9], [10],^14^]. Water quality criteria are contingent on natural conditions and may vary over time and across regions. Evaluating groundwater quality in mining locations is crucial to understanding the impact of mining activities and other anthropogenic influences on water quality [15]. Geochemical models are indispensable for understanding the interactions between rocks and water along hydrologic flow routes [8]. Groundwater physiochemical parameters indicate spatial differences in groundwater activity caused by rock-water interactions along flow routes. Geochemical modeling using NETPATH software serves as a vital tool for assessing groundwater quality, shedding light on key geochemical factors [16]. The minerals saturation index (SI) reflects various geochemical reactions, including rock-water interactions, dissolution reactions, salt solubility, precipitation, evapotranspiration, ion exchange, and anthropogenic activities [8,11,14,17]. In conjunction with a geochemical model, the inverse approach helps simulate the net geochemical mass balance, transfer, and reactions of potential minerals and gases in a groundwater environment [18]. A series of aqueous geochemical measurements were conducted to assess the degree to which reactions occur, utilizing saturation indices (SI) of the main mineral phases of the groundwater system. NETPATH model was used for this purpose [8,19]. The study's primary goal was to employ a geochemical model to evaluate the major geochemical processes influencing overall groundwater chemistry in the Quaternary aquifer in the Sukari Gold Mine region.

Besides groundwater contamination, the rapid growth of industries and cities, in developing nations also plays a role in worsening air quality, which poses health risks to the public. Operations such as manufacturing and transportation release substances like dust particles, nitrogen oxides, sulfur dioxide, and volatile organic compounds into the air, causing issues, heart problems, and worsening preexisting health conditions. Thus, it is crucial not only to monitor groundwater pollution but also to address concerns regarding air quality to protect the health of people residing in these areas [[20], [21], [22], [23], [24], [25]]. Surveys indicate a substantial gold deposit of around 7.7 million ounces in one section alone, making it a pivotal player in the region's mining landscape [1,26]. Located about 22 km southwest of Marsa Alam, SGM is strategically positioned in the central area of the Eastern Desert. Surrounded by mines of various minerals, the site incorporates diverse operations such as Open Pit Mines, Underground Mines, Processing Plants, Gold Rooms, Power Plants, and other project services. While contributing to the economy, mining activities introduce various hazards and emissions that can impact the well-being of workers in specific locations [27]. The historical significance of the Eastern Desert in gold mining dates back to 4000 B.C., with more than 90 sites across the Precambrian basement rocks. SGM is Egypt's first large-scale, modernized gold mine, reflecting the evolving economy's dynamics. However, activities such as open-pit and underground mining, processing, and power generation can pose hazards and emit pollutants affecting the work environment.

Therefore, the study's core objectives involve a comprehensive assessment of the designated area. Initially, we aim to (1) evaluate air and water quality, scrutinize risk exposure, and identify physical and chemical hazards in both work and ambient environments. (2) Subsequently, a modified simple model will be applied for risk assessment and comparative analysis of pollutant levels against national and international guideline values. Anticipated outcomes are significant: (3) giving recommendations and prescribing corrective actions based on the thorough evaluation of air and water quality, risk exposure, and identified hazards. This overarching objective seeks to eliminate workplace hazards, ensuring a secure working environment for all workers and mining staff. Insights gained from this study are positioned to guide the formulation of effective recommendations, contributing to establishing a safer and healthier workplace.

## 2 Thewhich is materials and methods

### 2.1 Site description

**Fig. 1:**
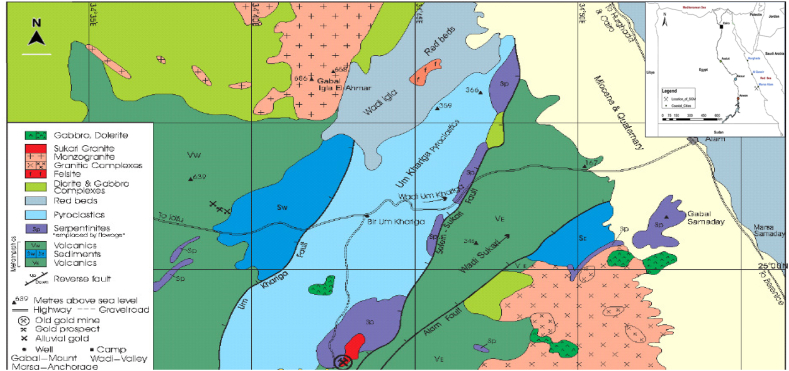
Geological and location of Study Area.

The SGM is situated in the Eastern Desert of Egypt, precisely at coordinates 24o 56′ 50″ N, 34o 42′ 27″ E, in the southeast region. It is positioned approximately 700 km away from Cairo and 25 km from the Red Sea Coastal City. Covering an expansive area of 160 square kilometers, as illustrated in Fig. 1, SGM commenced its production operations in 2009 and has experienced remarkable growth, achieving an annual output of 500 koz (103 ounces) by 2017. In 2015, the Sukari Processing Plant witnessed a significant upswing in ore-rock processing, handling a total of 10.6 million tons (Mt). This marked a substantial 26 % increase compared to the previous year, with the processing volume at 8.4 million tons in 2014. Consequently, this surge in processing led to the production of 439 kg of gold, a notable increase from the 377 kg produced in 2014. The total gold reserve estimated for the mine is an impressive 13 million ounces (370 tons). Over the years, the workforce at SGM has expanded from 900 employees in 2011 to 1500 in 2019. The mining techniques employed at SGM encompass a combination of traditional open-pit and underground methods to extract gold deposits. High-grade gold ore is sourced from underground operations, complemented by significant contributions from the open-pit mine to the overall production process. The extracted ore undergoes standard processing steps, including crushing, grinding, and flotation. The precious metal extraction involves a series of steps where gold is leached from the concentrate in a dilute cyanide solution, forming bonds with activated carbon. Subsequent separation processes efficiently extract gold metal from the carbon. This complex process adheres to established methodologies outlined by Razanamahandry [28], providing a robust framework for effective gold extraction at SGM. The hydroclimatological features of the Sukari Gold Mine region reflect the challenges associated with water availability and variability in arid environments. Effective water management strategies, including groundwater exploration, water conservation measures, and adaptation to climate change, are essential for the sustainable development of mining activities and local communities in the region. From 1984 to 2020, when examining Marsa Alam through Landsat images, it was discovered that urban development had grown by 3.71 km and green spaces had expanded by 0.81 km. This emphasizes the need to incorporate land cover and land use plans to promote the city's development [29].

### 2.2 Geological setting

The geological setting of the Sukari Gold Mine, depicted in Fig. 1, unfolds against the backdrop of the Arabian-Nubian Shield, a formation dating back 900-650 million years. The rock sequence at Sukari, an integral part of this shield, comprises calc-alkaline igneous rocks and metal sediments [30]. The Um Khariga Metapyroclastics, situated west of the Sukari granitoid and surrounding serpentinite, and the Sukari Metavolcanics are separated by this district-scale system, extending 25 km and passing immediately to the east of Sukari. Vail [31] dated these rocks to 770-660 million years ago, and regional metamorphism has transformed them into the mid-upper greenschist facies.

The Sukari felsic porphyry outcrop encompasses andesite flows, serpentinites, and associated volcaniclastic sediments, primarily tuffs and epiclastics. This geological feature spans 2.3 km and elevations from 100 to 600 m. The Sukari region is geographically divided into four zones from south to north: Amun, Ra, Gazelle, and Pharaoh, as outlined in the Sukari Gold Project Technical Report from March 2012. This zoning system facilitates a systematic understanding of the geological features and mineralization patterns within the Sukari Gold Mine area [32].

**Fig. 2:**
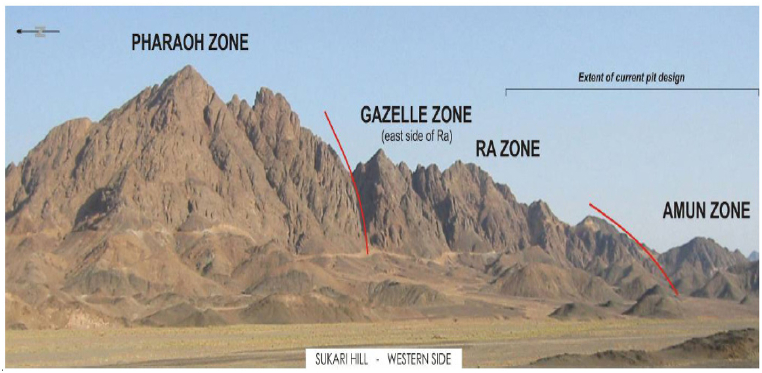
Sukari Hill with designated geographical zones (looking to the SE).

The hanging wall series at the Sukari Gold Mine comprises Serpentinite, Metaconglomerate, lesser fine-grained metal sediments, minor basalt, and porphyry dykes or sills, as illustrated in Fig. 2. The porphyry dykes in the hanging wall series are likely to be genetically and temporally similar to the significant Sukari Porphyry. On the eastern slope of Sukari Ridge, individual stratigraphic units, usually comprising more competent units in serpentinite, can be traced over tens of meters.

#### 2.2.1 Air and water quality monitoring and measurements

**Fig. 3:**
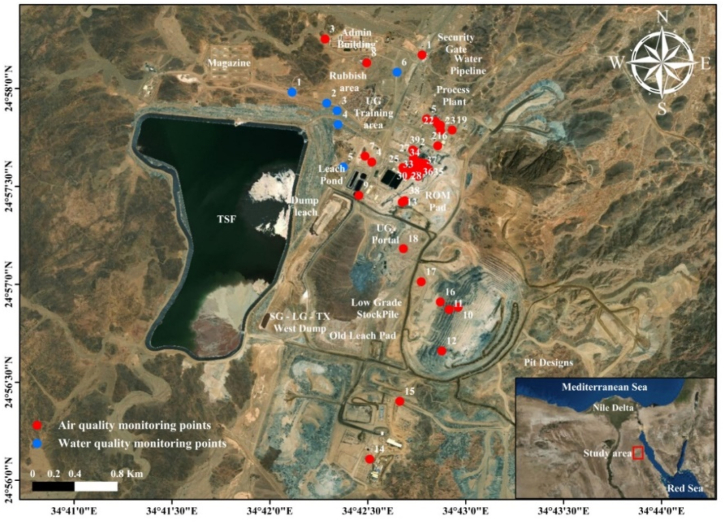
Air and water quality monitoring points Map-SGM site.

SGM initiated a comprehensive Environmental Monitoring program, meticulously examining various facets such as noise levels, airborne particulate matter (PM_10_), and gas emissions across its workplaces. This extensive endeavor covered 39 locations, including working areas, ambient environments, offices, control rooms, and a workers' camp. The primary objective was to thoroughly assess and monitor environmental conditions in these diverse settings, ensuring a comprehensive understanding of the air and atmospheric quality within the SGM. Specific Sukari Air Monitoring Points, strategically identified as illustrated in Fig. 3, played a crucial role in this evaluation. In parallel, to gauge the impact of mining operations on groundwater quality, SGM strategically excavated six boreholes near the tailing storage facility (TSF), constituting Sukari Water Monitoring Points (Fig. 3). The selection of boreholes around the project was thoughtfully determined to provide suitable area spacing based on the groundwater gradient. These six sampling sites for groundwater were instrumental not only in measuring physicochemical parameters such as pH, electrical conductivity, alkalinity, turbidity, suspended solids, and CN− but also in analyzing eleven major elements (Na^+^, K^+^, Ca^2+^, Mg^2+^, NH_4_^+^, Cl−, NO_3_^−^, HCO_3_^−^, SO_4_^2−^, PO_4_^3−^, F^−^, and Cl^−^) and heavy metals (Pb, Zn, Cd, Fe, Cu, As, and Mn). The investigation into air quality in workplaces encompassed measuring meteorological data, noise levels, dust emissions (PM_10_), and gases emissions. This comprehensive approach aimed to ensure a holistic understanding of the environmental impact of mining activities at the Sukari Gold Mine. The harmful gases assessed included CO, SO_2_, NO_2_, HCN, and NH_3_. The commitment to Environmental occupational health and safety (OHS) is evident in the implementation of training and monitoring programs at SGM, which are aimed at developing and increasing employee awareness of health, safety, and environmental (HSE) matters. This proactive approach ensures a holistic consideration of health, safety, and environmental factors in the mining operations at SGM.

#### 2.2.2 Air monitoring and analytical measurements methods

The study area's research strategy was successfully executed by employing high-standard equipment and devices for the necessary measurements in air quality monitoring for both workplace and ambient environments. Before the measurements, these instruments were meticulously calibrated to ensure their accuracy, and samples were taken to validate the precision of the measurements, in strict adherence to established standards.

In the case of water quality monitoring, a rigorous approach was adopted. Samples were meticulously collected, and the analysis was outsourced to an external laboratory. This external laboratory was chosen to guarantee that the highest analysis and procedure standards were followed. By outsourcing the analysis to a specialized facility, the study aimed to ensure the accuracy and reliability of the sample analysis results, meeting the stringent requirements set by industry standards. This meticulous approach enhanced the credibility and validity of the research findings in assessing air and water quality in the study area.

#### 2.2.3 Water sampling and analytical methods

The process of sampling groundwater within the gold mining plant area was executed meticulously, involving the careful collection from observation wells with depths less than 50 m because the depth at which groundwater samples were collected for our study. This depth range was selected based on prior research indicating that groundwater in our study area typically occurs at depths shallower than 50 m. Refined plastic bottles were used for this purpose, and the collected data spanning from 2013 to 2019 underwent a rigorous analytical examination. To maintain the integrity of the samples and prevent contamination, the collection bottles were pre-rinsed with deionized water. Each site contributed two samples: the first for major ions analyses and the second for heavy metals analyses, the latter being acidified with nitric acid to achieve a pH below 2. All samples were diligently preserved at temperatures below four °C until the analytical processes were conducted, following standardized procedures. Subsequently, the groundwater samples, filtered through 0.45 mm polypropylene filter membranes, underwent chemical analysis utilizing a variety of apparatus and techniques. Field measurements of physical parameters, including temperature, pH, total dissolved solids (TDS), and electrical conductivity (EC), were conducted using a portable calibrated salinity multi-parameter instrument (Hanna HI 9811-5). Major ions such as Ca^+2^, Mg^2+^, Cl^−^, HCO_3_^−^, and CO_3_^−2^ were subjected to volumetric titration, following a standard analytical method (APHA 2012). Flame photometry (PFP7 U.K.) was employed to analyze K+ and Na + ions, while UV/Visible spectrophotometry was used for SO_4_^2−^ and NO_3_^−^ analysis. Silica (SiO_2_) analysis was carried out through ion chromatography, and trace elements (Al, F, Fe, Mn) were measured using a spectrometer. The analytical results, presented in detail in Table 5, were complemented by an analysis of the mineralogical composition of aquifer materials derived from collected core samples. The presence of Al_2_O_3_, CaO, Fe_2_O_3_, SiO_3_, K_2_O, and MgO was identified in these materials. For groundwater data evaluation, statistical analysis was performed using SPSS Inc. software in Chicago, IL, USA. This comprehensive approach ensures a thorough understanding of groundwater quality in the study area, encompassing major ions, heavy metals, and various physical parameters.

**Table 1:** Noise levels (2013:2019) average reading results.

**ID/No.**	Location Name	Results(dB)	EEL4/94 Limits/8hrs
**Control Rooms &Offices**			
**#2**	Security Office	50.8	>70 dB (High) <70 dB (Low)
**#3**	Camp Area	58.2	
**#7**	Clinic	53.0	
**#8**	New Admin Office	69.5	
**#23**	Power Plant Office	69.8	
**#37**	Plant Control room	69.0	
**#38**	Crusher Control Room	62.4	
**#39**	Process and Mining Office	55.3	
**Work Environment**			
**#1**	Main Road	50.1	>90 dB (High) <90 dB (Low)
**#4**	Batch Plant Area	71.0	
**#5**	LV Workshop	75.2	
**#6**	Warehouse	56.2	
**#9**	Mining Check Point	64.0	
**#10**	Drill Pattern	**91.0**	
**#11**	Digging Face	82.2	
**#12**	Blast Pattern	76.4	
**#13**	Room Pad	69.5	
**#14**	Waste Dump-Grade Control Area	65.6	
**#15**	Waste Dump-Pit Containers Offices	60.4	
**#16**	Underground Area 1(740 Access)	**91.4**	
**#17**	Underground Area 2 (Tagboard 770)	**102.5**	
**#18**	Underground Area 3	89.5	
**#19**	Mobile Maintenance Workshop	76.6	
**#20**	Area In front of the power plant	85.6	
**#21**	Power Generators House	**107.0**	
**#22**	Power station stacks Area	**95.6**	
**#24**	Inside Laboratory (Sample Preparation)	67.4	
**#25**	Outside laboratory	78.8	
**#26**	Carbon Regeneration area	77.1	
**#27**	Air Compressor Area	**100.4**	
**#28**	Plant Workshops	72.1	
**#29**	Mills Area	**97.8**	
**#30**	Cyanide Area	80.2	
**#31**	Floatation Tank Area	80.6	
**#32**	Gold Room	61.7	
**#33**	Carbon Leaching Tank Area 1	85.4	
**#34**	Carbon Leaching Tank Area 2	85.9	
**#35**	At Regrind Area	79.2	
**#36**	At Lime Area	84.3

**Table 2:** PM_10_ dust (2013:2019) average reading Results-SGM site.

**ID/No.**	Location Name	PM_10_ Results	EEL4/94 Limits
**AMBIENT ENVIRONMENT (Time exposure 24 h)**			
**#1**	Main Road	85	>150 μg/m^3^(High) <150 μg/m^3^(Low)
**#3**	Camp Area	93	
**Work Environment (Time Exposure 8 Hrs.)**			
**#2**	Security Office	1.26	>3 mg/m^3^(High) <3 mg/m^3^(Low)
**#4**	Batch Plant Area	0.58	
**#5**	LV Workshop	0.77	
**#6**	Warehouse	0.85	
**#7**	Clinic	0.09	
**#8**	New Admin Office	0.08	
**#9**	Mining Check Point	0.98	
**#10**	Drill Pattern	1.51	
**#11**	Digging Face	0.59	
**#12**	Blast Pattern	0.39	
**#13**	Room Pad	0.25	
**#14**	Waste Dump-Grade Control Area	0.37	
**#15**	Waste Dump-Pit Containers Offices	0.47	
**#16**	Underground Area 1(740 Access)	**4.6**	
**#17**	Underground Area 2 (Tagboard 770)	**3.8**	
**#18**	Underground Area 3	**3.6**	
**#19**	Mobile Maintenance Workshop	0.4	
**#20**	Area In front of the power plant	0.37	
**#21**	Power Generators House	0.99	
**#22**	Power station stacks Area	0.99	
**#23**	Power Plant Office	0.89	
**#24**	Inside Laboratory (Sample Preparation)	**4.2**	
**#25**	Outside laboratory	1.98	
**#26**	Carbon Regeneration area	0.45	
**#27**	Air Compressor Area	0.86	
**#28**	Plant Workshops	0.99	
**#29**	Mills Area	0.37	
**#30**	Cyanide Area	0.20	
**#31**	Floatation Tank Area	0.27	
**#32**	Gold Room	0.99	
**#33**	Carbon Leaching Tank Area 1	0.12	
**#34**	Carbon Leaching Tank Area 2	0.18	
**#35**	At Regrind Area	0.89	
**#36**	At Lime Area	1.10	
**#37**	Plant Control room	0.20	
**#38**	Crusher Control Room	0.25	
**#39**	Process and Mining Office	0.35	

#### 2.2.4 NETPATH software description and assumption

The NETPATH software operates on the principles of geochemical mass-balance reactions, utilizing net geochemical mass-balance reactions between initial and final water along the hydrologic flow path [19]. NETPATH input parameters include constraints (solutes) and phases (minerals or gases), as outlined in Table 3, Table 4s. Prior to running the model, users can select inputs for dissolution and precipitation of phases, along with calculations for water mixing, evaporation, and dilution. The input data for the geochemical model encompass major mineral phases and constraints. The software evaluates the tendencies of Quaternary aquifer groundwater to precipitate or dissolve minerals, considering the saturation indices (SI) of hypothetical minerals in groundwater along the hydrologic flow path. Calculations include the number of reactants dissolved in water samples (in mmol/kg of H_2_O) and the number of products deposited from the solution. Additionally, the equilibrium degree concerning the dissolution/precipitation of a specific mineral can be determined using the saturation index (SI) according to Formula 2 [8,16]. This comprehensive approach offers valuable insights into the geochemical processes occurring in the groundwater system.

(2) SI = Log [IAP/K_sp_]

Where, The Ion Activity Product (IAP) and Solubility Product (K_sp_) are crucial parameters in understanding the dissolution and precipitation tendencies of minerals in a solution. The Saturation Index (SI), representing the logarithmic ratio of IAP to K_sp_, provides valuable insights into the saturation state of the solution. Negative SI values indicate sub-saturation, signifying the potential dissolution of minerals into the groundwater. Conversely, positive SI values suggest supersaturation, indicating the propensity of minerals to precipitate from the groundwater. A SI value of zero implies an equilibrium state between minerals and the solution, where dissolution and precipitation are balanced [8,[33], [34], [35]]. This information is essential for evaluating the dynamic equilibrium between minerals and groundwater in the geochemical system.

**Table 3:** Gasses emissions, Hl, and risk rating at site locations.

ID/No.	Location Name	CO	SO_2_	NO_2_	HCN	NH_3_	PM_10_	HI	Risk Rating
Work Environment EEL Limit (PPM)		25	3	2	4.7	25	3 mg/m3	>1 High <1 Low	Low(L) Medium(M) High(H)
Ambient Environment EEL Limit(μg/m^3^)		400	350	NIL	NIL	NIL	150		
Ambient Environment (Time exposure 24 h)									
#1	Main Road	0.27	0.1	0.1	0	0.1	85	0.0015	L
#3	Camp Area	0.1	0	0	0	0	93	0.0003	L
Work Environment (Time Exposure 8 Hrs.)									
#2	Security Office	0.24	0	0	0	0	1.26	0.01	L
#4	Batch Plant Area	0.1	0.1	0.6	0	0	0.58	0.34	L
#5	LV Workshop	0.72	0.1	0.4	0	2.75	0.77	0.37	L
#6	Warehouse	0.36	0	0	1	0	0.85	0.23	L
#7	Clinic	0.1	0	0	0	0	0.09	0.00	L
#8	New Admin Office	0.5	0	0	0	0	0.08	0.02	L
#9	Mining Check Point	0.1	0	0.1	0	1	0.98	0.09	L
#10	Drill Pattern	1.2	0	0.1	0	0	**1.51**	0.10	**H**
#11	Digging Face	0.8	0	0.1	0	0	0.59	0.08	L
#12	Blast Pattern	1.3	0	0	0	1	0.39	0.09	L
#13	Room Pad	0.35	0	0	0	0	0.25	0.01	L
#14	Waste Dump-Grade Control Area	0.92	0	0	0	0	0.37	0.04	L
#15	Waste Dump-Pit Containers Offices	0.12	0	0	0	0	0.47	0	L
#16	Underground Area 1(740 Access)	**6**	0.37	1.9	**7**	23.67	**4.6**	**3.75**	**H**
#17	Underground Area 2 (Tagboard 770)	**3.91**	0.23	1.8	**6**	15.7	**3.8**	**3.04**	**H**
#18	Underground Area 3	**2.8**	0.12	1.6	**5**	7	**3.6**	**2.30**	**H**
#19	Mobile Maintenance Workshop	2.9	0.1	0	0	0	0.4	0.15	L
#20	Area In front of the power plant	0	0.1	0.1	0	4.67	0.37	0.27	L
#21	Power Generators House	**2.9**	0.1	0.1	0	10	0.99	**0.60**	L
#22	Power station stacks Area	**2.8**	0.1	0.1	0	4	0.99	0.36	**H**
#23	Power Plant Office	0.1	0	0	0	0	0.89	0.00	L
#24	Inside Laboratory (Sample Preparation)	0.5	0.1	0.1	0	1	**4.2**	0.14	**H**
#25	Outside laboratory	0.6	0	0.1	0	1	1.98	0.11	L
#26	Carbon Regeneration area	2.2	0.1	0.1	0	1	0.45	0.21	L
#27	Air Compressor Area	0.1	0.1	0	0	2	0.86	0.12	**H**
#28	Plant Workshops	2.1	0.1	0.3	0	0	0.99	0.27	L
#29	Mills Area	1.5	0.1	0.5	0	2	0.37	0.42	**H**
#30	CN Area	0.1	0	0.3	1	3	0.20	0.49	L
#31	Floatation Tank Area	1.37	0	0.9	4	1	0.27	**1.40**	L
#32	Gold Room	0.47	0.1	0.9	0.1	2.8	0.99	0.64	L
#33	Carbon Leaching Tank Area (1)	2.1	0.1	1.9	**5.5**	15.5	0.12	**2.86**	**H**
#34	Carbon Leaching Tank Area (2)	**3.9**	0.1	0.7	5.4	14	0.18	**2.25**	**H**
#35	At Regrind Area	2.1	0.1	0.5	0	4	0.89	**0.53**	L
#36	At Lime Area	0.1	0	0.1	0	1	**1.10**	0.09	**M**
#37	Process Plant Control	2.1	0.1	0.2	0	1	0.20	0.26	L
#38	Crusher Control Room	0.1	0.1	0.2	0	0	0.25	0.14	L
#39	Process & Mining Office	0.1	0	0.1	0	1	0.35	0.09	L

**Table 4:** High/medium risk site locations at SGM site.

No.	Location Name	HI	Risk Rating		High-Risk Parameters	Control Measures
1	Drill Pattern	0.10	6	H	Noise, PM_10_	Every worker at these places must wear appropriate PPE and an HCN alarm. Gas detectors in areas with high HCN
2	Underground Area 1(740 Access)	3.75	6	H	Noise, PM_10_ CO, HCN	
3	Underground Area 2 (Tagboard 770)	3.04	6	H		
4	Underground Area 3	2.30	6	H		
5	Power Generators House	0.60	6	H	Noise	
6	Power station stacks Area	0.36	6	H	Noise	
7	Inside Laboratory (Sample Preparation)	0.14	6	H	PM_10_	
8	Air Compressor Area	0.12	6	H	Noise	
9	Mills Area	0.42	6	H	Noise	
10	Carbon Leaching Tanks Area (1)	2.86	24	H	HCN	
11	Carbon Leaching Tank Area (2)	2.25	24	H	HCN	
12	At Lime Area	0.09	23	M	PM_10_

**Table 5:** Analytical results of physicochemical parameters for the collective groundwater samples in the study area.

Year	No.	K^+^	Na^+^	Mg^2+^	Ca^2+^	NO_3_^−-^	Cl^−^	SO_4_^2--^	HCO_3_^−^	Fe	Mn	Al	F
2013	**Min**	1	52	180.8	340	0	1150	400	32.9	0	0	0.02	0.02
	**Max**	52	683	815.4	1125	1.21	3300	1200	79	0.2	0.1	1.25	1.3
	**Mean**	15	190.33	362.02	588.3	0.3517	1856.9	566.67	50.017	0.12	0.0511	0.7617	0.7833
	**SD**	20.209	242.5	242.69	282.3	0.4587	817.24	320.42	16.37	0.074833	0.0412	0.4176	0.5805
2014	**Min**	7	203.9	94	260	0	1183	200	30.8	0	0	0	0
	**Max**	60	811	630.2	1823.3	0.51	4845	910	330.1	1	1.01	1	1.01
	**Mean**	19.133	522.08	386.62	1006.1	0.1783	3172.2	464.33	197.02	0.339444	0.2672	0.3394	0.2672
	**SD**	20.263	250.36	222.3	582.05	0.2412	1339.7	240.16	112.94	0.450779	0.4291	0.4508	0.4291
2015	**Min**	25	173	34.8	123.6	0	1240	61	99	0	0	0	0.01
	**Max**	2635	7739.5	1983.5	3420	64.1	18156	5612	3419	2.11	0.865	0.85	1.8
	**Mean**	504.61	2748.9	684.45	1506	20.458	7145.5	1702.2	837.33	1.001667	0.5579	0.3483	0.6867
	**SD**	1046.2	2912.6	681.32	1467	25.558	6937.5	2035	1274.2	0.871376	0.348	0.3218	0.6811
2016	**Min**	52	683	180	340	0.1	1890	380	79	0.06	0.06	0.02	0.02
	**Max**	412.5	5460	1485.5	3745	9.03	16634	7590	184.5	10.02	1.44	0.1	1.02
	**Mean**	163.67	2948.5	929.83	1986.8	2.405	8899.4	2242.4	129.58	2.076667	0.5767	0.0467	0.1867
	**SD**	133.22	2004.1	548.04	1340.7	3.3455	5655.5	2691.7	41.1	3.94008	0.4885	0.0413	0.4082
2017	**Min**	44.6	704.9	49.4	491.8	0.1	1641.5	350.5	118	0.1	0.06	0	0
	**Max**	283.5	9686.9	1338	4450	27.05	22326	3049	870	2.3815	2.46	0.1	1.01
	**Mean**	190.57	5740.1	814.68	2502.1	14.033	14228	1727.1	286.2	1.0065	0.9533	0.0333	0.1733
	**SD**	97.688	3838.8	450.02	1374.7	12.118	8380.6	1003.3	287.94	0.94797	0.9595	0.0516	0.41
2018	**Min**	20.8	2591	829.6	1662.4	0.1	12973	1650	101.1	0.1	0.181	0	0
	**Max**	445	12828	2156.5	3925	45.1	25397	5250	208	4.95	1.3	0.1	0.5
	**Mean**	143.73	7464.9	1599.1	2921.6	17.747	18042	3127.5	156.08	1.7045	0.585	0.0367	0.09
	**SD**	157.08	4184.2	515.29	881.36	17.681	4897.6	1633.8	43.745	1.706564	0.414	0.0497	0.2011
2019	**Min**	6.9	805	18.2	1090	0.14	7300	33	70	0.1	0.1	0.02	0.02
	**Max**	91.3	10920	1506.6	5450.9	9.2	28123	1861.7	228	2.82	22.6333	6.6333	12.2667
	**Mean**	47.633	5547.1	1094.6	3621.4	2.6117	16935	938.97	152.37	1.593111	9.1658	3.1478	6.6017
	**SD**	29.189	3488.1	551.12	1756.6	3.3064	7259.8	683.82	64.077	1.019396	10.0878	2.6558	5.3494

All parameters are expressed in mg/L.

#### 2.2.5 Hazard index (HI) and risk assessment models

Risk assessments play a crucial role in assisting SGM operators in identifying, prioritizing, and managing risks within their operations. The process of risk assessment entails determining the likelihood and seriousness of harm related to recognized risks. This information is then used to determine the overall level of risk. The goal is to provide a structured approach to decision-making, allowing SGM management and operators to allocate resources and implement safety measures where they are most needed. To enhance safety in mining workplaces, various tools and approaches are employed. The systematic approach known as Hazard Identification and Risk Analysis (HIRA). The primary goal of HIRA is to implement a formalized process for identifying hazards, assessing associated risks, and implementing effective control measures to enhance workplace safety. This commitment to safety is crucial in the mining industry, where the nature of operations involves inherent risks that need to be carefully managed to protect workers and the surrounding environment.

#### 2.2.6 Hazard index (HI)

The HI (Formula 1) is a dimensionless value, and its interpretation is critical for understanding the cumulative health risk associated with exposure to multiple substances. If the HI is less than 1, it generally indicates a low risk. Conversely, values exceeding 1 suggest an increased potential risk, especially when approaching or surpassing the value of 1. The HI provides a comprehensive approach to assessing health risks associated with exposure to mixtures of harmful substances in gold production projects [9,36].

(1) HI=(C_1_ / TLV_1_) + (C_2_ / TLV_2_) + (C_3_ / TLV_3_) + … …. (C_n_ / TLV_n_) = 1

In the context of this formula, n represents the total number of substances concurrently measured in the same workplace. C1 denotes the concentration of substance 1, with TLV1 representing its Threshold Limit Value – Time-Weighted Average (TLV-TWA). This TLV corresponds to the concentration of a hazardous substance in the air averaged over an 8-h workday and a 40-h workweek. The same principle applies to C2,C3, …, and their respective TLVs (TLV2, TLV3, and so forth). If the HI is equal to or exceeds unity, it signifies that the combined exposure to these substances surpasses the permissible limit. This holds true even if the individual concentrations of these chemical substances are below their specified Time-Weighted Averages (TWAs).

#### 2.2.7 Risk management

In managing workplace risks, a qualitative method is employed for risk classification, as detailed in Table 1s. Furthermore, Risk Likelihood, as provided in Table 2s, serves as a valuable guide in this process. This methodology is crucial for assessing occupational health and mitigating adverse effects on employees within the workplace. The risk analysis model plays a crucial role in establishing recommendations and control actions required to remove or reduce hazards in the workplace. By qualitatively assessing risks and determining their likelihood, organizations can develop strategies to create a safer working environment. This involves identifying potential hazards, evaluating their severity, and assessing the probability of occurrence. The qualitative method likely involves categorizing risks based on their potential impact and the likelihood of occurrence. This categorization may include levels such as high, medium, and low risk. Tables 1s and 2s likely outline the criteria and guidelines for classifying risks and assessing their likelihood. Once risks are classified, appropriate recommendations and control measures can be implemented to effectively address and manage these risks. This proactive approach helps prevent accidents, occupational diseases, and other negative impacts on employee health and safety. Furthermore, the risk analysis model is instrumental in identifying and detecting unavoidable risks at their source. This information is valuable for decision-making and prioritizing actions to ensure a safer and healthier work environment for all employees.

#### 2.2.8 Data analyses and graphical approach

In the investigation, various tools and software were applied to analyze groundwater chemistry intricacies. The Piper diagram facilitated the visual categorization of diverse water types, while the Chadha diagram aided in discerning specific geochemical processes. Insights into the primary factors influencing groundwater chemistry were derived from the Gibbs diagram. The Geochemist's Workbench Student Edition 12.0 software was utilized for modeling and analyzing geochemistry, enabling the generation of diagrams based on collected water data to better understand the geochemical landscape. Additionally, the NETPATH software package v. 2.0 was employed to simulate environmental conditions using groundwater parameters, shedding light on the complexities of geochemical reactions occurring along the hydrologic flow path. Throughout the study, emphasis remained on equilibrium and SI to assess the stability of minerals in groundwater. These indices played a crucial role in unveiling the thermodynamic aspects of the aquifer, offering valuable insights into ongoing geochemical processes [19].

## 3 Results and discussion

### 3.1 Assessment of air quality

The primary objective of this research was to evaluate the air quality at the Sukari site and assess environmental pollution at SGM. The focus was on identifying potential environmental hazards and ecological risks and proposing necessary control measures to ensure a safe workplace environment, thus contributing to effective environmental protection measures at the site.

### 3.2 Meteorology

**Fig. 4:**
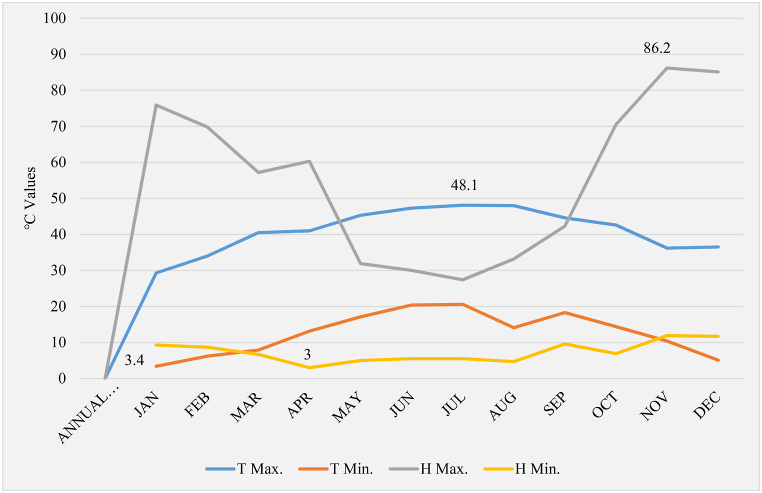
Annual temp.& humidity values (2013:2019) average - SGM site.

Meteorological data gathered from the weather station erected at the Sukari site provided insights into the weather conditions in the working area. The annual temperature and humidity data (Fig. 4) revealed significant variations. Recorded temperature values indicated the highest temperature at T Max. 48.1 °C and the lowest at T Min. 3.4 °C. Concurrently, humidity values during the same period showed the maximum humidity at H Max. 86.2 and the minimum at H Min. 3. These observations classified the Sukari Hill location under a hot desert climate category. Given these conditions, the Health, Safety, and Environment (HSE) department must address heat stress management during high temperatures. This was crucial to ensure the safety and well-being of both the work environment and the workers, particularly during the summer period.

### 3.3 Noise levels – decibels (dB)

**Fig. 5:**
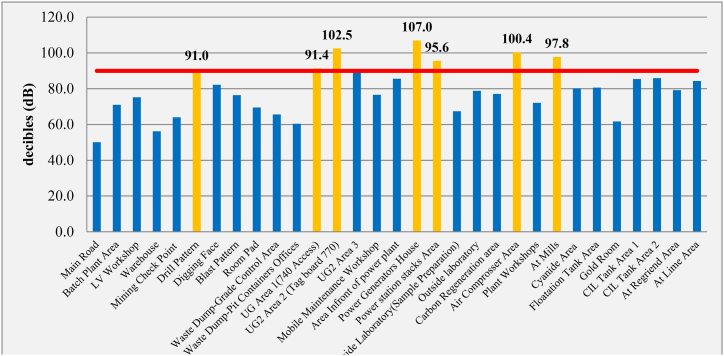
Noise levels (2013:2019) average - SGM work environment.

The as noise problem gained significant attention, especially in the mining sector where workers were exposed to high noise levels for extended periods, which could cause injury to their hearing and other senses. Staff members who disregarded workplace noise control standards might have experienced hearing loss due to loud exposure. To address this, noise assessment and control measures were imperative. The SGM site served as a noteworthy example of effective noise level management in the workplace. Fig. 5 shows the locations of the 39 places where the noise levels were carefully monitored.

**Fig. 6:**
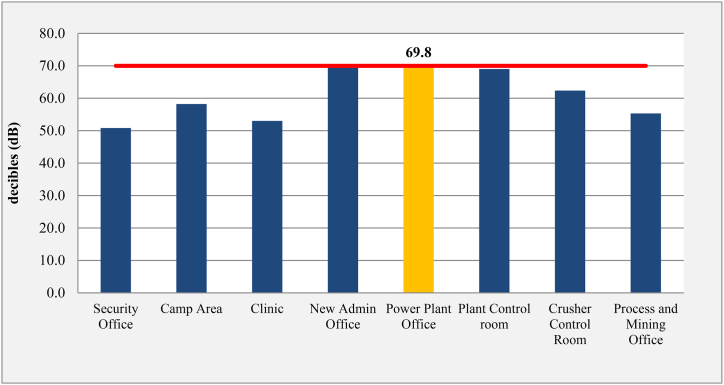
Noise levels (2013:2019) average - SGM control rooms & offices.

These locations included offices, control rooms, working and ambient settings, and camp areas. As seen in Fig. 6, the results indicated that the observed noise levels ranged from 50.1 dB to 107 dB. According to EEL4/94, the maximum noise level that could be heard in workingplaces during an 8-h workday was 90 dB. The highest noise levels were recorded in the Power Generators House (107 dB), UG2 Area 2 - Tagboard 770 (102.5 dB), Power station stacks Area (95.6 dB), Air Compressor Area (100.4 dB), At Mills (97.8 dB), Underground Area 1–740 Access (91.4 dB), and Drill Pattern (91 dB). Table 1 and Fig. 5 showed that seven workplaces exceeded this guideline. At these locations, control measures like lowering exposure limits and using PPE were implemented.

Furthermore, the EEL 4/94 recommendation value of 70 dB for an 8-h exposure was compared to the noise levels measured in control rooms, offices, and camp areas. The findings are shown in Table 5. All offices had noise levels measured, and they were all below the permitted threshold. The power plant office, at 69.8 dB, was the closest to the limit. It was advised always to keep this office door tightly closed, to place rubber insulators on the door edges correctly, and to think about using double glass to improve isolation effectiveness.

### 3.4 Dust emissions - particulate matters (PM_10_)

**Fig. 7:**
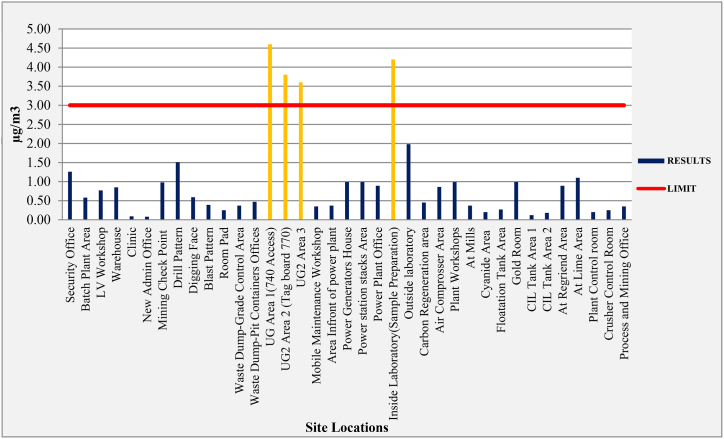
Particulate matters (PM_10_) -(2013:2019) average SGM work environment.

The significance of this endeavor cannot be overstated, as it is well-documented that individuals laboring in environments characterized by elevated dust concentrations, particularly in the mining industry, confront an escalated risk of developing respiratory ailments [37]. In response to this pressing concern, an exhaustive assessment of respirable dust (PM_10_) levels was conducted at 39 locations, encompassing both operational and surrounding areas. The findings, delineated in Table 2 and illustrated in Fig. 7, unveil a notable range in PM_10_ values, with the new admin office registering the minimum at 0.08 mg/m3, while four locations, including Underground Access No 740, UG2 Area 2 (Tagboard 770), UG2 Area 3, and the laboratory (Sample Preparation room), recorded maximum values of 4.6 mg/m3, 3.8 mg/m3, 3.6 mg/m3, and 4.2 mg/m3, respectively.

In adherence to the EEL 4/94 guidelines for an 8-h exposure, most recorded values fall below the permissible threshold of 3 mg/m3, except for the four locations mentioned above. Crucial measures to mitigate PM_10_ levels have been enacted in these sites, encompassing water depression, secure closure of airborne sources from machinery, and the utilization of appropriate PPE. Within the ambient environment, the EEL 4/94 guideline designates 150 mg/m3 as the permissible value for PM_10_ for a 24-h exposure, and the measured concentrations in these areas conform to this permissible limit.

It is imperative to underscore that the dust sources in these locations stem from land erosion by the wind and are not directly linked to mining activities, since these places are at least one km upwind of the mine. To further curtail dust dispersion, a recommendation is made to strategically plant a curtain of trees in the upwind vicinity of the camp and main roads. This strategic environmental intervention holds paramount importance, aiming to enhance the overall air quality and diminish the impact of dust in both operational and surrounding areas.

### 3.5 Gas emissions (CO, SO_2_, NO_2_, HCN, NH_3_)

The operational activities at SGM led to the emission of various gases, including CO, SO_2_, NO_2_, NH_3_, and HCN, affecting both the workplace and the ambient environment. Elevated concentrations of these gases, surpassing their Threshold Limit Values (TLV) or EEL4/94 limits, posed potential health risks to on-site workers. Concentrations of these gases were systematically measured at 39 locations, covering diverse activities such as open-pit mining, underground mining, the processing plant, and the gold room. While some sites showed no detectable gas emissions, others simultaneously detected one or more gases, each with varying concentration levels. Detailed results of gas emissions measurements can be found in Table 3.

### 3.6 Carbon Monoxide (CO)

The highest Carbon Monoxide (CO) concentration, as presented in Table 3, was observed in the Underground areas and the CIL Tanks Area. Importantly, these concentrations remained within the limits established by EEL4/94 standards.

### 3.7 Sulfur (SO_2_) and Nitrogen Dioxides (NO_2_)

The SGM deposit shows low Sulfur Dioxide (SO_2_) and Nitrogen dioxide (NO_2_), as indicated in Table 3. Crucially, the detected levels did not exceed the limits specified by EEL4/94 standards. The origin of SO_2_ in the SGM project is attributed to the liberation of sulfur from the host minerals of gold. Additionally, the release of Nitrogen Dioxide (NO_2_) from the power plant remains below its TLV-TWA (Threshold Limit Value - Time-Weighted Average) in all identified locations**.**

### 3.8 Hydrogen cyanide (HCN)

Hydrogen Cyanide (HCN) is identified as a toxic gas produced during gold cyanidation [38,39]. Occupational exposure to Hydrogen Cyanide has been detected in Carbon in Leaching Tanks (CIL Tanks 1 & 2), with concentrations of 5.5 ppm and 5.4 ppm, respectively, as outlined in Table 3. In Underground areas, HCN was identified at concentrations of 7 ppm in Underground Area 1 (740 Access), six ppm in Underground Area 2 (Tag Board 770), and five ppm in Underground Area 3. These Hydrogen Cyanide values surpass the limits set by EEL 4/94, prompting recommendations for workers in Underground and Carbon in Leaching Tanks locations. These include carrying dosage meters with alarms to detect high HCN concentrations, wearing a gas mask when concentrations exceed ten ppm, and evacuating the area when levels surpass 20 ppm. These measures are crucial for ensuring the safety of workers amidst potential gas exposures.

### 3.9 Ammonia (NH_3_)

Ammonia (NH_3_) has been detected in various workplaces and is associated with different mining activities. Table 3 indicates noteworthy concentrations in Underground, Gold Room, and Carbon in Leaching Tanks areas, each with distinct values. However, it is important to note that the concentrations in other locations are comparatively lower, and all measured levels remain below the ammonia Time-Weighted Average (TWA) limits of 25 ppm.

### 3.10 Hazard index (HI) and risk assessment rating model

Combined exposures to multiple gas emissions or mixtures necessitated special consideration when evaluating occupational health hazards. The HI was employed to calculate the relative safety of exposure to mixtures of harmful substances. For substances with similar toxicological effects, such as toxic gases in gold production projects, the HI was estimated using Formula 1 [36], and the results were provided in Table 3. If the HI equals or exceeds unity, the exposure to combined substances surpasses the limit, even if the individual concentrations of these chemicals or gas emissions are below their Time-Weighted Average (TWA). Several sites registered simultaneous detections of more than one gas emission in the investigated areas, designating them as high-risk areas, notably in Underground areas, as indicated in Table 3. While no individual gas surpasses its Threshold Limit Value-Time-Weighted Average (TLV-TWA), the estimated HI exceeds 1, signifying that the occupational hazard in these locations exceeds the permissible limit for combined exposure. Similar findings of HI greater than unity were observed in three other locations within the workplaces, specifically in Flotation Tanks and CIL Tanks 1&2. Control programs must be implemented in environments with HI > 1 to reduce gas concentrations to allowable limits.

In addition to HI, a risk assessment (Quantitative method Table 1s) was applied to calculate the risk rating for occupational health in each workplace. If the risk assessment of one or more gases indicates high risk, the workplace is considered high risk for worker health, necessitating control measures. For workplaces with moderate risk, interventions are required to prevent negative effects on workers. A case study of the modified risk assessment model was implemented for the workplace in SGM, with results recorded in the last column of Table 3. Among the workplaces, 27 were classified as Low Risk, one as Medium Risk, and 11 as High Risk. Notably, some high-risk workplaces also had HI values exceeding unity, and the medium-risk site had HI values surpassing half. While HI determines whether a workplace poses an occupational hazard to workers' health, it does not specify which gas emissions contribute to this hazard. The risk assessment model comprehensively explains the sources of such hazards. Therefore, high and medium-risk locations should be classified as the most hazardous areas in the workplace. Workers in these areas must wear the required PPE during work hours, as outlined in the high/medium-risk Table 3s below and Risk Assessment locations.

## 4 Assessment of water quality

### 4.1 Hydrogeology

**Fig. 8:**
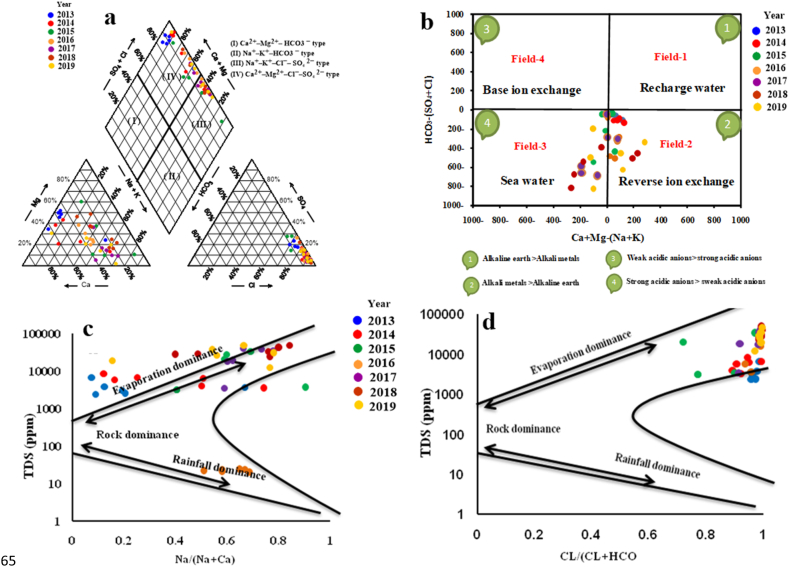
Piper Tri Linear Diagram (a), Chadha diagram (b), Gibbs diagram (c and d).

In a broader context, the Sukari region faces arid conditions with sporadic yet intense rainfall, potentially resulting in significant water runoff from the Wadi into the pit. The projected minimal and localized water inflows into the pit underscore the critical need for this study. Utilizing Piper's trilinear diagram [5]serves as a crucial tool to represent the groundwater types within the sampled aquifer visually (see Fig. 8a). The chemical analysis of groundwater samples unveils two distinct types: Na–K–Cl–SO_4_ and Ca–Mg–Cl–SO_4_. These findings play a pivotal role in identifying the sources of solutes, shedding light on the influences from diverse recharging sources such as surface canals, drains, irrigation return flow, anthropogenic activities, and the excessive use of fertilizers and pesticides [40,41]. The prevailing water types highlighted by the Piper diagram (Fig. 8a) indicate elevated salinity and the concluding phase of water evolution, coupled with saltwater intrusion. The application of CHADHA's classification to the collected groundwater samples, elucidated in Fig. 8b, serves as a crucial step in discerning hydrochemical processes and groundwater facies. Fields 2 and 3 within the CHADHA diagram align with the groundwater samples, revealing a reverse ion exchange mechanism in field two and a mixture with saline water in field 3. The correlation between the results from the Piper diagram and the CHADHA diagram (Fig. 8b) reinforces the significance of this study, affirming that the groundwater quality in the study area is primarily shaped by dissolved Na+ and K+, as well as reverse ion exchange processes between Ca^2+^ and Mg^2+^ within the clay aquifer matrix. This becomes particularly crucial considering the prevalence of saltwater intrusion and water–rock interaction in the predominantly Quaternary sediments of fluvial-lacustrine origin, comprising sand and gravel with silt and clay intercalations in the study area.

Gibbs diagram [7], constructed by plotting TDS vs. the ratios Na/(Na + Ca) and Cl/(Cl + HCO_3_), serves as a valuable tool to elucidate the key processes influencing groundwater chemical composition. The dispersion of groundwater points in the evaporation, runoff, and rock water domination areas is discerned from the plot of geochemical data on the Gibbs diagram (Fig. 8c and d). Evaluation based on the Gibbs diagram indicates that most groundwater samples fall within the rock-dominated zone, highlighting the significant impact of evaporation and rock-water contact on groundwater chemistry. The prevalence of rock dominance and prolonged interaction between rock and water substantially influence groundwater chemistry, as indicated by the results in (Table 5s). Moreover, the groundwater samples exhibit a subtle inclination towards the prevailing evaporation area, signifying elevated TDS and chloride concentrations resulting from the cumulative effects of seawater intrusion and aquifer materials. Consequently, groundwater samples from the rocky field tend to migrate towards the evaporation field. Human activities have contributed to degrading the original groundwater's consistency, transforming it into semi-salty water (brackish). The impact of reverse ion exchange processes on the evolution of groundwater content in the sample region is evident, aligning with prevailing water forms in the area, such as Na–K–Cl–SO_4_ and Ca–Mg–Cl–SO_4_ facies, as illustrated in the Piper diagram (Table 5). Thus, these two processes emerge as critical determinants shaping the chemical composition of groundwater in the region.

### 4.2 Geochemical stability

#### 4.2.1 Saturation indices

In this study, the NETPATH geochemical model was employed to evaluate mineral content [19], mineral saturation states, and groundwater's propensity to either precipitate or dissolve minerals [42]. Analyzing the saturation indices for the groundwater samples (Table 5s) and (Table 5). The model results revealed that groundwater samples from the year 2013 were undersaturated, and groundwater samples from the year 2013 were undersaturated concerning quartz, calcite, aragonite, talc, gypsum, and dolomite. The SI for quartz ranged from −1.406 to −0.932, with a mean of −1.214 and a standard deviation of 0.18. Calcite exhibited SI values ranging from −1.352 to −1.092, averaging −1.254, with a standard deviation of 0.108. Aragonite SI values ranged from −1.208 to −0.949, averaging −1.101, with a standard deviation of 0.108. Talc SI values ranged from −9.015 to −7.208, averaging −7.944, with a standard deviation of 0.686. Gypsum SI values ranged from −0.812 to −0.254, averaging −0.620, with a standard deviation of 0.224. Dolomite SI values ranged from −2.263 to −1.829, averaging −2.09, with a standard deviation of 0.165. These findings suggest that mineral equilibrium did not constrain the soluble components in groundwater, and mineral dissolution occurred as a result.

In 2014, the groundwater samples were found to be undersaturated regarding quartz and gypsum. The SI for quartz ranged from −1.315 to −0.408, with an average of −0.8632 and a standard deviation of 0.39743. Gypsum SI values ranged from −1.079 to −0.47, averaging −0.724, with a standard deviation of 0.24073. Aragonite SI values varied from −0.064 to 0.352, with an average of 0.1776 and a standard deviation of 0.164488. Talc SI values ranged from −0.216 to 2.976, averaging −1.2682, with a standard deviation of 1.22804. Calcite SI values ranged from 0.08 to 0.495, with a mean of 0.3512 and a standard deviation of 0.166297. Dolomite SI values ranged from 0.294 to 0.903, with an average of 0.5286 and a standard deviation of 0.27742.

Similarly, in 2015, the groundwater samples were undersaturated in quartz and gypsum. Quartz SI varied from −1.012 to −0.044, averaging −0.5446, with a standard deviation of 0.45915. Gypsum SI values ranged from −0.904 to 0.342, with an average of −0.2726 and a standard deviation of 0.59062. Aragonite SI varied from −0.201 to 0.452, with an average of 0.129 and a standard deviation of 0.276586. Talc SI values ranged from −0.461 to 4.623, averaging 2.414, with a standard deviation of 2.42367. Calcite SI values ranged from −0.057 to 0.595, with a mean of 0.2726 and a standard deviation of 0.27625. Dolomite SI values ranged from −0.394 to 1.795, averaging 0.7092, with a standard deviation of 0.92781.

In 2016, the groundwater samples remained undersaturated regarding quartz and gypsum. Quartz SI varied from −2.207 to −0.77, averaging −1.269, with a standard deviation of 0.55372. Gypsum SI values ranged from −0.542 to 0.585, averaging −0.0372 and a standard deviation of 0.43101. Aragonite SI varied from −0.293 to 1.141, averaging 0.6406, with a standard deviation of 0.59336. Talc SI values ranged from −7.176 to 7.692, averaging 1.9976, with a standard deviation 5.75146. Calcite SI values ranged from −0.149 to 1.285, averaging 0.7846 and a standard deviation of 0.5933601. Dolomite SI values ranged from −0.228 to 2.625, averaging 1.6426, with a standard deviation of 1.16989.

In 2017, the groundwater samples were undersaturated concerning quartz, aragonite, talc, and dolomite. Quartz SI ranged from −1.056 to −0.608, with an average of −0.8126 and a standard deviation of 0.22076. Aragonite SI varied from −0.907 to 0.384, averaging −0.117 and a standard deviation of 0.496561. Talc SI values ranged from −4.873 to 0.744, averaging −1.1904 and a standard deviation of 2.21067. Dolomite SI values ranged from −1.775 to 1.302, averaging −0.0196 and a standard deviation of 1.12705. Calcite SI values ranged from −0.763 to 0.527, with a mean of 0.0268 and a standard deviation of 0.4963085.

In 2018, all groundwater samples were observed to be undersaturated regarding quartz. Quartz SI varied from −0.962 to −0.315, with a mean of −0.544 and a standard deviation of 0.27599. Calcite SI values ranged from 0.236 to 1.164, averaging 0.6836 and a standard deviation of 0.3583494. Aragonite SI varied from 0.093 to 1.02, with a mean of 0.54 and a standard deviation of 0.358164. Talc SI values ranged from 2.081 to 5.318, with an average of 3.6798 and a standard deviation of 1.51004. Gypsum SI values ranged from −0.028 to 0.482, with an average of 0.1974 and a standard deviation of 0.

The and the geochemical model (NETPATH) geochemical model (NETPATH) reveal the dynamic interaction between rock and water in the aquifer system. In 2013, negative saturation index (SI) values for minerals such as quartz, calcite, aragonite, talc, gypsum, and dolomite indicate the groundwater's capacity to dissolve these minerals, allowing for increased concentrations of Ca, Mg, SO_4_, and SiO_2_ constituents. In 2014, favorable SI values for carbonate minerals (calcite, aragonite, and dolomite) in suggested their potential precipitation from groundwater, leading to a reduction in suggested their potential precipitation from groundwater, leading to reduction in suggested their potential precipitation from groundwater, leading to a reduction in reducing dissolved Ca and Mg ions. Negative SI values for gypsum indicate the groundwater's ability to dissolve this mineral, accommodating more Ca and SO_4_ constituents. Additionally, negative SI values for quartz imply the groundwater's capability to dissolve silicate minerals and tolerate higher SiO_2_ concentrations. The trend continues in 2015 and 2016, with negative SI values for quartz and gypsum, signifying their potential dissolution and increased tolerance for SiO_2_, Ca, and SO_4_ constituents. Positive SI values for carbonate minerals (calcite, aragonite, and dolomite) and talc indicate their propensity to precipitate from groundwater, depleting dissolved Ca and Mg ions. In 2017, quartz, aragonite, talc, and dolomite dissolution is evident, allowing for higher SiO_2_, Ca, and Mg concentrations. The precipitation of calcite and gypsum signals the loss of dissolved Ca and SO_4_ ions. In 2018, key processes includedindicated included quartz dissolution and the precipitation of calcite, aragonite, talc, gypsum, and dolomite, which indicated included quartz dissolution and the precipitation of calcite, aragonite, talc gypsum, and dolomite, indicating the depletion of Ca, Mg, and SO_4_ constituents. The trend continued in 2019, with the dissolution of quartz, talc, and gypsum, accommodating higher SiO_2_, Ca, and Mg constituents. Carbonate minerals with positive SI values, such as calcite, aragonite, and dolomite, contributed to the depletion of Ca and Mg in the groundwater. These findings provide valuable insights into the mineral dynamics within the aquifer system, shedding light on processes of dissolution and precipitation that impact the composition of groundwater constituents over time.

#### 4.2.2 Limitations of the research work

The study's limitations in the context of mining operations in Egypt might include challenges related to access to data or resources, regulatory constraints, limitations in technology or infrastructure, and potential environmental or social factors that could impact our research process or findings.

The future scope of the research could involve exploring advancements in mining technologies, conducting further environmental impact assessments, investigating sustainable mining practices, or examining the socio-economic implications of mining activities in Egypt. Additionally, there may be opportunities to collaborate with industry stakeholders, government agencies, or academic institutions to address emerging issues and drive innovation in the mining sector.

#### 4.2.3 Recommendations based on the study findings

**Noise Management:** Employees working in high-noise areas must consistently wear hearing protection.

To reduce noise exposure, implement screens, barriers, enclosures, and absorbent materials.

**Dust Control:** Workers in high-dust environments should always use respiratory masks.

Ensure adequate workplace ventilation and employ water dump trucks for dust suppression on roads.

Plant a curtain of trees in upwind locations to mitigate dust emissions.

**Gas Emissions:** Provide suitable PPE for workers in high gas emission areas. Install Hydrogen Cyanide (HCN) gas detectors at locations with high HCN concentrations.

Evacuate areas when gas levels exceed safety limits.

**Groundwater Quality Monitoring:** Establish continuous groundwater quality measurements and monitoring for all boreholes and wells around the Tailing Storage Facility (TSF).

Ensure ongoing assessment to prevent groundwater contamination resulting from mining operations.

These recommendations aim to enhance workplace safety, protect the environment, and ensure the sustainable operation of the mining site. Regular monitoring and adherence to safety protocols are essential to a comprehensive risk management strategy.

## 5 Conclusion

The effectiveness of industrial operations relies heavily on the meticulous identification, evaluation, and mitigation of potential hazards and associated environmental risks. This research offers a comprehensive examination of environmental management practices at the Sukari Gold Mine (SGM), situated in the southeastern desert of Egypt. Rigorous environmental assessments were conducted to evaluate air and water quality, identify hazards, and assess risks within the SGM premises. Air quality and noise levels were significantly measured at 39 distinct locations across the mine's operational areas. The findings revealed non-compliance with noise exposure regulations outlined in the Egyptian Environmental Law (EEL4/94), with the Power Generator House registering the highest noise levels at 107 dB. Remedial actions, such as implementing personal protective equipment (PPE) and strategies to reduce noise exposure, are being considered to address these elevated noise levels effectively. Measurements of particulate matter (PM10) and various noxious gases (e.g., CO, SO2, NO2, HCN, and NH3) were conducted in workplace and ambient environments. Elevated concentrations of PM10, particularly in underground areas, necessitated the implementation of water suppression techniques and enhanced PPE measures. While most gas emissions remained within regulatory limits, certain zones exhibited hydrogen cyanide (HCN) levels exceeding permissible thresholds, prompting the need for specific control measures. Utilizing hazard index (HI) and risk rating assessments, this study identified varying risk profiles across different workplaces within SGM, highlighting areas requiring targeted intervention. Additionally, an evaluation of water quality near a Tailing Storage Facility (TSF) was conducted to evaluate the impact of mining activities on groundwater quality. The study revealed that groundwater in the region belongs to the Na–K–Cl–SO_4_ and Ca–Mg–Cl–SO_4_ water classes, with potential degradation attributed to high mineralization processes induced by aquifer materials and seawater intrusion.

These findings highlight the importance of continuous monitoring, proactive control measures, and the implementation of environmental initiatives to ensure the sustainability of mining operations at the Sukari Gold Mine. Initial steps in securing the workplace and assessing ecological risks involve defining and analyzing hazards. Despite resource constraints, addressing all hazards remains critical, with prioritization guided by environmental measurements, hazard analysis, and risk assessment. This ensures that vital conditions are promptly addressed, followed by those with lower probabilities but potentially significant consequences. The study conducted at the SGM site, combined with HI and risk rating assessments, identified numerous high-risk conditions due to the lack of implemented control measures. To enhance workplace safety, particular attention should be paid to noise-generating processes, strictly enforcing PPE usage in high-risk areas. Additionally, careful planning of road spacing in mining areas, mainly underground locations, is essential for safe machine movement, including the installation of traffic signals and signage. Utilization of physicochemical parameters, geochemical simulation, statistical analysis, and GIS techniques emerges as a practical and efficient approach for groundwater quality assessment and monitoring.

mainly.

## Funding

This study was funded by 10.13039/501100002383King Saud University through Researchers Supporting Project number (RSP2024R133), King Saudia University, Riyadh, Saudia Arabia.

## Institutional review board statement

Not applicable.

## Informed consent statement

Not applicable.

## Data availability statement

All data are provided as tables and figures in the manuscript and supplementary material.

## CRediT authorship contribution statement

**Ahmed Ali El-Sayed M. Ata:** Writing – original draft, Software, Methodology, Conceptualization. **Mobarak H. Aly:** Writing – original draft, Resources, Methodology. **Hend Hussein:** Writing – original draft, Software, Conceptualization. **Mohamed Hamdy Eid:** Writing – review & editing, Writing – original draft, Visualization, Supervision, Software, Methodology. **Mostafa R. Abukhadra:** Writing – review & editing, Writing – original draft, Project administration, Methodology, Conceptualization. **Ahmed M. El-Sherbeeny:** Writing – review & editing, Writing – original draft. **Stefano Bellucci:** Writing – review & editing, Writing – original draft, Resources, Funding acquisition. **Mohamed Gad:** Writing – review & editing, Writing – original draft, Supervision, Software, Methodology.

## Declaration of competing interest

The authors declare that they have no known competing financial interests or personal relationships that could have appeared to influence the work reported in this paper.
