# Supplementary material for: Hydrogeochemical characteristics and air quality risks associated with gold mining operations in Egypt using geochemical modeling and risk indices
Source: Heliyon. 2024 May 22;10(11):e31086. doi: 10.1016/j.heliyon.2024.e31086 (PMC11145231; doi:10.1016/j.heliyon.2024.e31086)
Supplement: Multimedia component 1 [file mmc1.docx]

**Table 1s.** Qualitative Method for the Classification of Risks

| **Risk Rank Likelihood x Consequence** | **L1 Almost certain** | **L2  Likely** | **L3 Possible** | **L4 Unlikely** | **L5  Rare** |
| --- | --- | --- | --- | --- | --- |
| **C1 Catastrophic** | **1** | **2** | **4** | **7** | **11** |
| **C2 Major** | **3** | **5** | **8** | **12** | **16** |
| **C3 Moderate** | **6** | **9** | **13** | **17** | **20** |
| **C4 Minor** | **10** | **14** | **18** | **21** | **23** |
| **C5 Insignificant** | **15** | **19** | **22** | **24** | **25** |

| **Table 2s.** Risk Likelihood for guidance | | | | | | |
| --- | --- | --- | --- | --- | --- | --- |
| **Step 1: Assess the Likelihood** | | | | **Step 2: Assess the Consequences** | | |
| **L1** | Happens Every time we operate | Almost Certain | Common or repeating occurrence | **C1** | Fatality | Catastrophic |
| **L2** | Happens Regularly (often) | Likely | Known to have occurred” has happened” | **C2** | Permanent disability | Major |
| **L3** | Has happened (occasionally) | Possible | Could occur or heard of it happening | **C3** | Medical/ hospital or lost time | Moderate |
| **L4** | Happens irregularly (almost never) | Unlikely | No likely to occur | **C4** | First aid or no lost time | Minor |
| **L5** | Improbable  (never) | Rare | Practically impossible | **C5** | No injury | Insignificant |

| **RISK RATING** | |
| --- | --- |
| **High  Risk** | **1 to 6** |
| **Medium  Risk** | **7 to 15** |
| **Low  Risk** | **16 to 25** |
| **RISK RATING** | |
| **High  Risk** | **1 to 6** |
| **Medium  Risk** | **7 to 15** |
| **Low  Risk** | **16 to 25** |

**Table 3s.** List of Constraints for NETPATH

| **1: Carbon** | **2: Sulfur** | **3: Calcium** | **4: Aluminium** |
| --- | --- | --- | --- |
| **5: Magnesium** | **6: Sodium** | **7: Potassium** | **8: Chloride** |
| **9: Fluoride** | **10: Silica** | 11: Bromide | 12: Boron |
| 13: Barium | 14: Lithium | 15: Strontium | **16: Iron** |
| **17: Manganese** | 18: Nitrogen | 19: Phosphate | 20: Redox |
| 21: Carbon - 13 | 22:C - 14 | 23: Sulfur-34 | 24: Strontiun-87 |
| 25: Nitrogen - 15 | 26: Deuterium | 27: Oxygen - 18 | 28: Tritium |

Elements in bold are considered in the simulation

**Table 4s.** List of Phases for NETPATH

| 1:"CH_2_O"^+^ | **2: ALBITE+** | 3: ALUNITE | 4: ANALCIME^+^ |
| --- | --- | --- | --- |
| 5: ANNITE^+^ | **6: ANORTHITE+** | **7: ARAGONITE** | 8: AUGITE |
| 9: BARITE | 10: BIOTITE+ | 11: BRUCITE | **12: CALCITE** |
| 13: CELESTITE | 14:CH_4_ GAS | **15: CHLORITE+** | 16: CHRYSOTL^+^ |
| **17: CO2 GAS** | 18: CO2 – CH_4_ | 19: Ca - MONT | 20: DIOPSIDE+ |
| **21: DOLOMITE** | 22: EXCHANGE | 23: FLUORAP | **24: FLUORITE** |
| 25: FORSTERITE^+^ | 26: FeS | 27: FeII-Na | 28: GIBBSITE |
| 29: GEOTHITE | **30: GYPSUM** | 31:H2 GAS | **32: H_2_S GAS** |
| 33: HEMATITE | 34: HORNBLEND | 35: HYDROXAP | **36: ILLITE** |
| **37: K-SPAR^+^** | 38: K-MICA+ | 39: K-MONT | **40: KAOLINITE** |
| 41: LIGNITE^+^ | 42: MAGNESITE | 43: MAGNITITE | 44: MGO_2_-CAL |
| 45: MIRABILI | 46: MONT-FEL | 47: MONT-MAF | 48: Mg-MONT |
| 49: Mg/Na EX | 50: MgSiO_3_^+^ | 51: Mn (OH)_3_ | 52: MnO_2_ |
| 53: MnOOH | 54: N_2_ GAS | 55:NA-MONT | NACLINOP |
| 57: NH_3_ GAS | 58: NH_4_/CAEX | 59: Na-MONT | 60: Na_2_SO_4_ |
| **61: NaCl** | 62: NaHCOL | 63: O_2_ GAS | 64: PHLOGOPI |
| 65: PLAGAN25^+^ | 66: PLAGAN30^+^ | 67: PLAGAN33+ | 68: PLAGAN38^+^ |
| 69: PLAGAN45^+^ | 70: PREHNITE^+^ | **71: PYRITE** | 72: RHODOCHR |
| 73: SEPIOLITE | 74: SIDERITE | 75: STRENGITE | 76: STRONITE |
| 77: SELVITE | **78: SiO_2_** | **79: TALC** | 80: VIVANITE |
| 81: WITHRIT | 82:"OTHERS" |  |  |

Phases in bold are considered in the simulation

**Table 5s.** Saturation Indices Values for Groundwater Samples

| Year | No | Quartz | Calcite | Aragonite | Talc | Gypsum | Dolomite |
| --- | --- | --- | --- | --- | --- | --- | --- |
| 2013 | **1** | -1.406 | -1.208 | -1.352 | -9.015 | -0.812 | -2.263 |
|  | **2** | -1.252 | -1.182 | -1.326 | -7.208 | -0.254 | -2.143 |
|  | **3** | -1.315 | -1.136 | -1.279 | -7.977 | -0.781 | -2.05 |
|  | **4** | -0.932 | -1.032 | -1.176 | -7.51 | -0.68 | -2.174 |
|  | **5** | -1.165 | -0.949 | -1.092 | -8.013 | -0.574 | -1.829 |
|  | **min** | -1.406 | -1.208 | -1.352 | -9.015 | -0.812 | -2.263 |
|  | **max** | -0.932 | -0.949 | -1.092 | -7.208 | -0.254 | -1.829 |
|  | **mean** | -1.214 | -1.1014 | -1.245 | -7.9446 | -0.6202 | -2.0918 |
|  | **SD** | 0.18058 | 0.1085 | 0.1087 | 0.6861 | 0.2249 | 0.16545 |
| 2014 | **1** | -0.408 | 0.31 | 0.166 | 1.619 | -0.532 | 0.294 |
|  | **2** | -0.508 | 0.495 | 0.352 | 2.976 | -0.47 | 0.903 |
|  | **3** | -1.315 | 0.42 | 0.127 | 1.54 | -0.781 | 0.35 |
|  | **4** | -0.92 | 0.08 | -0.064 | 0.422 | -1.079 | 0.348 |
|  | **5** | -1.165 | 0.451 | 0.307 | -0.216 | -0.758 | 0.748 |
|  | **min** | -1.315 | 0.08 | -0.064 | -0.216 | -1.079 | 0.294 |
|  | **max** | -0.408 | 0.495 | 0.352 | 2.976 | -0.47 | 0.903 |
|  | **mean** | -0.8632 | 0.3512 | 0.1776 | 1.2682 | -0.724 | 0.5286 |
|  | **SD** | 0.39743 | 0.166297 | 0.164488 | 1.22804 | 0.24073 | 0.27742 |
| 2015 | **1** | 0.044 | 0.311 | 0.168 | 3.917 | 0.123 | 0.391 |
|  | **2** | -0.158 | 0.044 | -0.1 | 0.028 | -0.02 | -0.394 |
|  | **3** | -1.012 | -0.057 | -0.201 | -0.461 | 0.342 | 0.21 |
|  | **4** | -0.799 | 0.595 | 0.452 | 4.623 | -0.904 | 1.795 |
|  | **5** | -0.798 | 0.47 | 0.326 | 3.963 | -0.904 | 1.544 |
|  | **min** | -1.012 | -0.057 | -0.201 | -0.461 | -0.904 | -0.394 |
|  | **max** | 0.044 | 0.595 | 0.452 | 4.623 | 0.342 | 1.795 |
|  | **mean** | -0.5446 | 0.2726 | 0.129 | 2.414 | -0.2726 | 0.7092 |
|  | **SD** | 0.45915 | 0.27625 | 0.276586 | 2.42367 | 0.59062 | 0.92781 |
| 2016 | **1** | -2.207 | -0.149 | -0.293 | -7.176 | 0.585 | -0.228 |
|  | **2** | -0.77 | 1.148 | 1.004 | 4.676 | 0.177 | 2.223 |
|  | **3** | -1.018 | 1.285 | 1.141 | 7.692 | -0.215 | 2.625 |
|  | **4** | -1.083 | 1.095 | 0.951 | 4.428 | -0.191 | 2.356 |
|  | **5** | -1.267 | 0.544 | 0.4 | 0.368 | -0.542 | 1.237 |
|  | **min** | -2.207 | -0.149 | -0.293 | -7.176 | -0.542 | -0.228 |
|  | **max** | -0.77 | 1.285 | 1.141 | 7.692 | 0.585 | 2.625 |
|  | **mean** | -1.269 | 0.7846 | 0.6406 | 1.9976 | -0.0372 | 1.6426 |
|  | **SD** | 0.55372 | 0.5933601 | 0.59336 | 5.75146 | 0.43101 | 1.16989 |
| 2017 | **1** | -0.608 | -0.763 | -0.907 | -4.873 | 0.064 | -1.775 |
|  | **2** | -1.056 | 0.211 | 0.067 | -0.872 | -0.317 | 0.261 |
|  | **3** | -0.696 | 0.269 | 0.125 | 0.233 | 0.333 | 0.342 |
|  | **4** | -1.048 | 0.527 | 0.384 | 0.774 | -0.075 | 1.302 |
|  | **5** | -0.655 | -0.11 | -0.254 | -1.214 | 0.061 | -0.228 |
|  | **min** | -1.056 | -0.763 | -0.907 | -4.873 | -0.317 | -1.775 |
|  | **max** | -0.608 | 0.527 | 0.384 | 0.774 | 0.333 | 1.302 |
|  | **mean** | -0.8126 | 0.0268 | -0.117 | -1.1904 | 0.0132 | -0.0196 |
|  | **SD** | 0.22076 | 0.4963085 | 0.496561 | 2.21067 | 0.23661 | 1.12705 |
| 2018 | **1** | -0.449 | 0.236 | 0.093 | 2.081 | 0.215 | 0.534 |
|  | **2** | -0.318 | 0.461 | 0.317 | 2.658 | -0.028 | 0.791 |
|  | **3** | -0.962 | 1.164 | 1.02 | 5.256 | 0.061 | 2.437 |
|  | **4** | -0.676 | 0.865 | 0.722 | 3.086 | 0.482 | 1.812 |
|  | **5** | -0.315 | 0.692 | 0.548 | 5.318 | 0.257 | 1.742 |
|  | **min** | -0.962 | 0.236 | 0.093 | 2.081 | -0.028 | 0.534 |
|  | **max** | -0.315 | 1.164 | 1.02 | 5.318 | 0.482 | 2.437 |
|  | **mean** | -0.544 | 0.6836 | 0.54 | 3.6798 | 0.1974 | 1.4632 |
|  | **SD** | 0.27599 | 0.3583494 | 0.358164 | 1.51004 | 0.19638 | 0.78468 |
| 2019 | **1** | -0.861 | 0.125 | -0.019 | -1.035 | 0.166 | 0.02 |
|  | **2** | -0.986 | 0.136 | -0.008 | -1.863 | -0.099 | 0.144 |
|  | **3** | -1.027 | 1.338 | 1.194 | 7.843 | -0.662 | 2.488 |
|  | **4** | -0.817 | -1.374 | -1.517 | -10.347 | -1.748 | -4.146 |
|  | **5** | -0.989 | 0.767 | 0.624 | 2.55 | -0.265 | 1.681 |
|  | **min** | -1.027 | -1.374 | -1.517 | -10.347 | -1.748 | -4.146 |
|  | **max** | -0.817 | 1.338 | 1.194 | 7.843 | 0.166 | 2.488 |
|  | **mean** | -0.936 | 0.1984 | 0.0548 | -0.5704 | -0.5216 | 0.0374 |
|  | **SD** | 0.09134 | 1.0128816 | 1.012634 | 6.67065 | 0.74849 | 2.56024 |

Positive sign (+): precipitation of mineral (m. mole /Kg). Negative sign (−): dissolution of mineral (m. mole/Kg)

| **No.** | **Equipment** | **Parameter measured** | **Photo** |
| --- | --- | --- | --- |
| **1** | Name: **Sound level meter (Noise)**  Type: Testo AG Germany  Model: Testo 815  Manufacture: Germany  Serial Number: 30810585/011 | Noise Intensity Level(dB) | **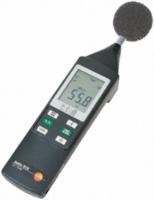** |
| **2** | Name: **DUST TRAK II Aerosol Monitor**  Type: 8530Dust Trak II Desktop  Model: 8530/8531/8532  Manufacture: USA  Serial Number: 8530111606 | PM10 | **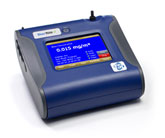** |
| **3** | Name: **V RAE**  (Multi Gas Monitor HCN, CO, NO_2_, NH_3_…)  Type: V RAE  Model: PGM-7800&7840  Manufacture: USA  Serial Number: 174-500523 | HCN, CO, NO_2_, NH_3_…) | **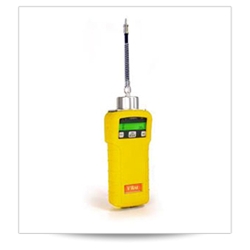** |
| **4** | Name: **Q RAE II**  (Multi Gas Detector H_2_S, O_2_, SO_2_…)  Type: QRAE II  Model: PGM-2400-P  Manufacture: USA  Serial Number: 020-1201-5B0 | SO_2_ | **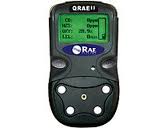** |
| **5** | Name: **Precision Weather Station**  Type: VANTAGE PRO  Model: 2001 \| VANTAGE PRO  Manufacture: USA  Serial Number: 6162C-Cabled | 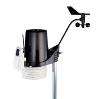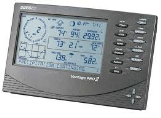  Temperature &Humidity Values | |

**Equipment, Devices, Sampling and Analytical Methods**
